# Risk Factors for Persistence or Recurrence of High-Grade Cervical Squamous Intraepithelial Lesions

**DOI:** 10.1590/0100-6991e-20233537-en

**Published:** 2023-11-18

**Authors:** Dulcimary Dias Bittencourt, Rita Maira Zanine, Ana Paula Martins Sebastião, Carmen Marcondes Ribas

**Affiliations:** 1 - Universidade Federal do Paraná, Tocoginecologia - Curitiba - PR - Brasil; 2 - Universidade Federal do Paraná, Patologia - Curitiba - PR - Brasil; 3 - Faculdade Evangélica Mackenzie do Paraná - Curitiba - PR - Brasil

**Keywords:** Biomarkers, Cervix, Neoplasms, Recurrence, Surgical Procedures, Neoplasia Intraepitelial Cervical, Conização, Recidiva

## Abstract

**Objectives:**

*t*o evaluate whether the colposcopic lesion size , age, kind of surgery, the status of the surgical margins and the expression of the p16 and Ki-67 immunomarkers are risk factors for persistence or recurrence of the lesion.

**Methods:**

a cross-sectional, observational, retrospective study of patients submitted to cold knife conization (CKC) or the loop electrosurgical excision procedure for cervical intraepithelial neoplasia 2 or 3. The colposcopic lesion size, age, surgical method, involvement of the surgical margins, and p16/Ki-67 immunomarker expression were analyzed in relation to lesion persistence and recurrence.

**Results:**

seventy-one women were treated with cold knife conization and 200 were treated with loop electrosurgical excision. Of these, 95 had cervical intraepithelial neoplasia 2, 173 had cervical intraepithelial neoplasia 3, 183 had free surgical margins, 76 had compromised margins, and 12 showed damage by processing artifact or fragments. Among the 76 cases with positive margins, 55, 11, and 10 showed endocervical margin involvement, ectocervical margin involvement, and both endocervial and ectocervical margin involvement, respectively. Of the 264 followed-up patients, 38 had persistent or recurrent disease. A multiple logistic regression indicated that positive endocervical margins are the only independent risk factor for the persistence/recurrence of cervical intraepithelial neoplasia. No significant association was identified between the colposcopic lesion size, age, surgery type, or p16/Ki-67 immunomarker expression and lesion persistence or recurrence.

## RESUMO

**Objetivos:**

avaliar se o status das margens, idade, tamanho da lesão colposcópica, tipo de cirurgia e expressão dos marcadores p16/Ki-67 são fatores de risco na persistência ou recidiva da LIEAG.

**Métodos:**

um estudo de corte transversal, observacional com coleta de dados retrospectivos de pacientes submetidas a conização a frio (CF) ou exérese da zona de transformação por cirurgia de alta frequência EZT por NIC2/3. Foram analisados os seguintes fatores em relação a persistência ou recidiva: comprometimento das margens, idade, tamanho da lesão, tipo de cirurgia e coexpressão dos imunomarcadores p16 e Ki-67.

**Resultados:**

271 mulheres tratadas com CF (71) e EZT (200), onde 95 apresentavam NIC 2 e 173 NIC 3, 183 apresentaram margens cirúrgicas livres, 76 comprometidas e 12 prejudicadas por artefatos ou fragmentação. Das 76 pacientes com margens comprometidas, 55 foram endocervical, 11 ectocervical e 10 ambas as margens. Das 264 pacientes que tiveram seguimento, 38 persistiram ou recidivaram a doença. A regressão logística múltipla indicou ser a margem endocervical comprometida o único fator independente de risco de persistência/recorrência da NIC (p<0,001). Não houve associação significativa entre a idade, o tamanho da lesão colposcópica, o tipo de cirurgia e a expressão dos imunomarcadores p16/Ki-67 e a persistência ou recorrência da doença.

**Conclusão:**

entre os fatores estudados associados com persistência ou recorrência, somente a margem endocervical comprometida provou ser significativamente um fator risco para persistência ou recorrência da lesão.

## INTRODUCTION

Cervical cancer is the third most common malignant tumor in the female population excluding non-melanoma skin cancer, behind only breast and colorectal cancer, and is the fourth leading cause of women’s deaths from cancer. It is responsible for the deaths of 311,000 women/year in the world and for 6,526 deaths in Brazil in 2018, with an estimated 16,710 new cases in 2020^1^.

Different treatments have been proposed over the years, including hysterectomy, conization, and currently, loop electrosurgical excision procedure (LEEP) is used more frequently, although it is associated with an increase in the number of fragments removed, which limits interpretation and increases the number of compromised margins^2^.

The correct assessment of the lesion, such as its exact location and extension, are important factors associated with selecting appropriate treatment. Additionally, the lesion size will determine the excision size and reflect the compromised margins.

Many are the considered risk factors for lesion persistence or recurrence after treatment, such as age, type of surgery, glandular involvement, colposcopic lesion size, immunosuppression, involved endocervical margin, and others^4^ ^,^ ^5^.

Biomarkers have recently emerged and are being used to help clinicians screen, detect, diagnose, and assess the prognosis of intraepithelial lesions. The expressions of the biomarkers p16 and Ki-67 suggest dysregulation of the cell cycle by HPV and are associated with lesion severity^6^ ^,^ ^7^.

This study explored the following potential factors for intraepithelial lesion recurrence: colposcopic lesion size, age, surgical method, compromised surgical margins, p16 expression, and Ki-67 expression of patients followed-up for a long period.

## METHODS

This is a cross-sectional, observational study with retrospective data collection, analyzing 360 medical records of women undergoing LEEP or cold knife conization (CKC) from January 2005 to July 2017 at the Hospital de Clínicas - UFPR - and subjected to immunohistochemical evaluation for p16 and Ki-67.

We included all patients who underwent the first LEEP or CKC procedure at the Hospital de Clínicas with a surgical specimen diagnosis of Cervical Intraepithelial Neoplasia (CIN) 2 and 3.

We excluded patients with autoimmune diseases or those with any immunodeficiency, histopathological results of the conization product with cervicitis, CIN 1, adenocarcinoma, and microinvasive or invasive carcinoma.

A total of 271 patients met the inclusion criteria, whose slides and paraffin blocks were retrieved to perform the Tissue Microarray Technique (TMA).

The CKC or LEEP procedures were all performed at the institution, by residents supervised by staff with extensive experience in surgical techniques.

The colposcopic lesion size was obtained by evaluating the drawing of the colposcopy in the medical records and counting the number of quadrants of the cervix affected by the lesion. As described by Lowers et al.^8^, we divided the cervix like a clock into four quadrants, the first would be between 12 and 3 o’clock, the second between 3 and 6, the third between 6 and 9, and the last between 9 and 12. We considered a small lesion to be one that affects one quadrant, a medium one that affects two, and a large lesion one that affects three or four quadrants.

The lesion is considered recurrence when appearing six months after the surgical procedure used for treatment, and persistence, when diagnosed before six months after treatment. For this study, we will use the term “recurrence”, regardless of the time in which the lesion appeared, due to the difficulty in knowing the exact moment^9^.

### Immunohistochemistry

We prepared 105 reading slides for p16 and 113 for Ki-67. The whole processing happened in the automated platform Ventana Benchmark Ultra^TM^, using the Antibodies p16 (Ventana clone E6H4, prediluted) and Ki-67 (Ventana, clone 30-9, prediluted).

### Immunomarker readings

The immunoreactivity of p16 and that of Ki-67 were evaluated according to the number of cells expressing labeling with differing intensity. Immunoreactivity was divided into the following three groups: 1, weak intensity; 2, moderate intensity; and 3, strong intensity. The thickness of the epithelium stained was divided into the following three levels: 1, lower one-third of the epithelium; 2, lower two-thirds of the epithelium; and 3, full thickness of the epithelium.

The project was approved by the Ethics Committee of Faculdade Evangélica Mackenzie Paraná, under number 55675716.7.0000.0103, and was developed at the Complexo Hospital de Clínicas, Universidade Federal do Paraná, Curitiba, PR, Brazil, under number 55675716.7.3001.0096.

### Statistical analysis

We described quantitative variables by means, standard deviations, medians, minimum and maximum values. We presented categorical variables as frequencies and percentages. We assessed the association between two categorical variables using the Chi-square test. We compared more than 2 groups in relation to quantitative variables with the one-way analysis of variance (ANOVA) model or the Kruskal-Wallis non-parametric test. To analyze the time until recurrence, we fitted Cox Regression models and estimated Hazard Ratio values, with 95% confidence intervals. To evaluate factors associated with the reports’ results, we fitted Logistic Regression models and estimated odds ratio values, with 95% confidence intervals. Values of p<0.05 indicated statistical significance. For multiple comparisons after ANOVA and Kruskal-Wallis tests, p values were Bonferroni-corrected. The data were analyzed using the Stata/SE v.14.1 software. StataCorp LP, USA.

## RESULTS

We included 271 women between the ages of 17 and 67 in the study. To analyze factors associated with recurrence, we excluded seven cases that were lost to follow-up.

Lesion size at colposcopy: Among the 271 cases, 11 (4.1%) had no lesions, in 69 (25.5%) the lesions were considered small, in 122 (45%) medium, and in 69 (25.5%), large.

Type of surgery: 71 (26.2%) underwent cold conization (CKC) and 200 (73.8%) underwent excision of the transformation zone with high frequency wave surgery (LEEP).

Lesion degree: 95 (35.1%) had CIN 2 and the remaining 176 (64.9%) had CIN 3.

Margins: In 183 cases (67.5%), free margins were reported, 76 (28%) had margins compromised by the lesion, and in 12 cases (4.4%) the margins were compromised by fragmentation artifacts. Of the 76 involved margins, 55 (72.4%) occurred in the endocervical portion, 11 (14.5%) in the ectocervical one, and 10 cases (13.2%) in both margins. These results are shown in Table 1.

**Table 1:** Age, lesion size , type of surgery, histological report, and margin status.

Variable	n	Classification	Result*	
Age (years)	271		35.5 ± 10.0	34 (17 - 67)
Size	271	No lesion	11	4.1%
		Small	69	25.5%
		Medium	122	45.0%
		Large	69	25.5%
Surgery	271	0-LEEP	200	73.8%
		1-CKC	71	26.2%
Report	271	CIN 2	95	35.1%
		CIN 3	176	64.9%
Margin	271	Free	183	67.5%
		Compromised	76	28.0%
		Fragmented/coagulated	12	4.4%
Compromised margin site	76	Endocervical	55	72.4%
		Ectocervical	11	14.5%
		Both	10	13.2%

Immunohistochemical reaction for p16: Of the 271 cases, we performed reactions in 105. Regarding the intensity of p16 staining, eight cases (7.6%) were mild, 32 (30.5%) moderate, and 65 (61.8%) were strong. As for the immunostained epithelial thickness, of the 105 cases, seven (6.7%) stained 1/3 of the epithelium, 36 (34.3%) stained 2/3, and 62 (59%), the entire epithelium.

Immunohistochemical reaction for Ki-67: Of the 271 cases, 113 reactions were performed. Regarding Ki-67 staining intensity, three cases (2.7%) were mild, 24 (21.2%) were moderate, and 86 (76.1%) were strong. As for the immunostained epithelial thickness, of the 113 cases, six (5.3%) occurred in 1/3 of the epithelium, 48 (42.5%) in 2/3 of the epithelium, and 59 (52.2%) occurred throughout the epithelium. These results are shown in Table 2.

**Table 2:** Results of immunostaining with p16 and Ki-67.

Variable	n	Score	n	%
p16 intensity	105	1	8	7.6
		2	32	30.5
		3	65	61.8
p16 grade	105	1	7	6.7
		2	36	34.3
		3	62	59.0
Ki-67 intensity	113	1	3	2.7
		2	24	21.2
		3	86	76.1
Ki-67 grade	113	1	6	5.3
		2	48	42.5
		3	59	52.2

Of the 264 cases with follow-up, 38 (14.4%) had recurrence, with a mean time of 22.7 ± 14.7 months. By the fifth year, 82.4% of patients were disease-free, and after the tenth year, 81%.

As for the colposcopic lesion size and recurrence, we observed that no case of lesion not visible at colposcopy recurred, small lesions recurred in 11.8%, medium sized lesions in 15.3%%, and large ones, in 17.9%, a gradual increase but without the possibility of carrying out a statistical study due to the lack of value in the group without injuries. To enable the statistical calculation, we grouped the cases of no injuries and small injuries, there being no statistical significance (Table 3).

**Table 3:** Recurrence according to lesion size.

Size	n	%	Size	n	p	95% CI
No lesion	0	0	No lesion/Small	8		
Small	8	11.55				
Medium	18	15.3	Medium	18	0.255	1.62(0.71-3.73)
Large	12	17.9	Large	12	0.123	2.02(0.83-4.95)

* Mann-Whitney non-parametric test, p<0.05.

When evaluating the association between age and lesion recurrence, we observed that the average age of women who had recurrence was 36.9 ± 9.6 years, with no statistical difference when compared with the age of patients who did not recur. Likewise, when we divided patients aged 35 years or over and those aged under 35 years, there were 17 patients in the first age group and 21 in the second, this difference not being significant.

Regarding the type of surgery and recurrence, most recurrences occurred in high-frequency surgeries, 37 of the 38 cases of recurrence (97.3%), but statistical calculation was unfeasible due to low frequency in the cold conization category.

When analyzing the association between the quality of the margins and recurrence, we found that the margins, when compromised by a lesion, have a significant association with its recurrence, especially the endocervical margin (Table 4).

**Table 4:** Recurrence according to compromised endocervical and ectocervical margins and both.

Local	Free	178	13 (7.3%)		
	Compromised endocervical	55	19 (34.6%)	<0.001	5.24 (2.59 - 10.6)
	Compromised ectocervical	11	2 (18.2%)	0.232	2.48 (0.56 - 11.0)
	Both	10	3 (30.0%)	0.032	3.96 (1.13 - 13.9)

* Mann-Whitney non-parametric test, p<0.05

There was a statistical association between compromised margins and type of surgery (Table 5).

**Tabela 5:** Type of surgery and margins.

	Classification	n	Surgery		p*
			LEEP	CKC	
Margin	Free	183	121 (66.1%)	62 (33.9%)	<0.001
	Compromised	76	67 (88.2%)	9 (11.8%)	

*Fisher’s exact test, p<0.05.

As for the association of p16 immunohistochemical reaction and recurrence, mild reaction obtained a recurrence rate of 12.5%, moderate p16 reaction of 22.6%, and strong reaction, 17.2% of recurrence. In the analysis of stained epithelial thickness, scales with value 1 had 14.3% recurrence, value 2 had 14.7% recurrence, and value 3 showed 21.7% recurrence, although there was no significant difference.

Regarding the Ki-67 reaction, 33.3% recurrence was associated light staining, 8.7% with moderate staining, and 20% with strong staining; in the evaluation of the stained epithelial thickness scale, we observed recurrence of 16.7% in value 1, 14.9% in value 2, and a higher, 20.7% recurrence for value 3, though without statistical significance.

## DISCUSSION

Much has been studied about the reasons for recurrence of cervical intraepithelial lesions after treatment, including the size of the colposcopic lesion, age, margins involvement, histological grade, glandular involvement, and immunosuppression^4^ ^,^ ^5^ ^,^ ^10^ ^,^ ^11^.

Recurrence occurs on average in 20.6% and can vary from 4.6 to 48%, which is an undesirable result both for doctor and patient, the former feeling that treatment was insufficient and the latter probably having to undergo a new procedure^4^ ^,^ ^5^ ^,^ ^9^. In our study, we observed a recurrence rate of 14.4%, below the average reported in the literature. Brockmeyer et al.^5^ have found a 48.1% recurrence rate, with 75% of recurrences occurring in the first year.

We observed a recurrence time interval of 22.7 months, 81% of patients being disease-free at the tenth year. Serati et al.^10^ found similar figures, with a mean recurrence time of 26.7 months, and with 75.4% of patients disease-free in the fifth year of follow-up, while Lili et al. found a longer average time to recurrence, of 46.5 months, explained by the diagnosis of an adenocarcinoma at 59.2 months^12^.

Patients must have adequate follow-up after treatment. Although some services use the HPV test^13^, we follow patients with cytology and colposcopy, and it is noteworthy that we had a small loss to follow-up, of 2.5%, as we actively searched for patients; in the literature, the lowest loss to follow-up found was by Gardeil et al., with 6.2%^14^.

Studies suggest that the size of the lesion should be evaluated in the management of CIN. Despite being a poorly discussed subject in the literature, it is considered a risk factor for lesion progression and recurrence, since false negative cytologies occur more commonly in small lesions. In addition, the exact location and extent of the lesion is a crucial factor in choosing the treatment, CKC or LEEP^14^ ^,^ ^15^.

In our study, the size of the Colposcopic lesions did not statistically significantly predict recurrence. However, recurrence trends were 11.8%, 15.3%, and 17.9% for small, medium, and large lesions, respectively. When the lesion was not visualized, no recurrence was observed. Colposcopy was indicated by repeated altered cytology test results.

Kawano et al.^16^ stated that the lesion involving more than two quadrants is a risk factor for compromised margins and consequently for recurrence, although we must emphasize that the visualization of the quadrants by the professional is subjective. In a report made by Hopman et al 1995^17^, only in 68% of the time there was interobserver agreement regarding the number of quadrants affected by the lesion.

The age of patients is also considered a risk factor for recurrence. Menopause induces atrophy of the endometrium and endocervix, which causes the transformation zone to retract into the canal, thus causing the lesion to establish itself within the canal^16^.

Zhu et al. observed age over 35 years as the only isolated factor for recurrence, but they selected 275 patients with compromised margins within a population of 4,336 LEEP procedures^18^.

Our study did not indicate an association between recurrence and patient age. This can be explained by the fact that we chose CKC every time the junction was not visualized. Therefore, a greater volume of the cervix was removed.

During our study, 97.3% of relapses occurred after LEEP; however, a statistical analysis was not possible because of the low relapse rates of patients treated with CKC. Similarly, El-nashar et al.^19^ reported more than twice as many relapses with LEEP.

As already mentioned, CKC removes a much larger piece of tissue, thus reducing the risk of compromised margins and consequently recurrence, this technique is preferred in cases of suspected glandular lesion or microinvasion, and in cases in which the squamoucolumnar junction is not visible^2^ ^,^ ^3^. 

We observed a significant value of compromised margins in LEEP, of 88.2%, versus 11.8% in CKC. Other authors also found values more than double the compromise on the LEEP margins, such as Chen et al.^20^ and Murta et al.^3^, who found 33.3% of compromised margins in LEEP and 24.9% in CKC. However, the metanalysis from Li, Chen, and Jiang^21^ obtained no significant difference between the two techniques.

The greater compromise in the LEEP margins is due the loops used for the procedure often not covering the width of the lesion, which is why it is important to choose the correct surgical method, because as stated by Ayhan^22^, compromised margins are better predictors of persistent or residual disease, which represents a problem for doctors and patients when planning follow-up and future therapy.

A series of factors are related to compromised margins, including age, size of the lesion, type of surgery, and surgeon’s training level^20^.

In the literature, the rate of surgical margins’ compromise of cervix specimens is quite variable, ranging from 6% to 49%, with an average of around 25%, which is in agreement with our reports^9^ ^,^ ^13^ ^,^ ^23^ ^,^ ^24^.

Our study found 28% of compromised margins, 67.5% of free margins, and 4.4% of the remaining margins were impaired for reading, findings similar to those in the literature, as shown by Lili et al.^12^, who studied 569 LEEP and 235 CKC procedures and found 28% compromised margins. Bittencourt et al.^23^, in a series of 118 LEEP, reported 11.8% of compromised margins and 2.5% of margins impaired for reading, the low frequency of compromised margins being justified by the procedures being performed by a single professional, with extensive experience in the LEEP technique, as well as extensive knowledge of the lower genital tract pathology.

Margin involvement has an impact on lesion recurrence, especially when this involvement is at the endocervical margin. In our analysis, the compromised endocervical margin was responsible for 34.6% of recurrences, while in free margins recurrence occurred in 7.3%^5^ ^,^ ^9^ ^,^ ^13^ ^,^ ^14^ ^,^ ^22^. De Mello e Silva et al.^4^ showed that in women with recurrence the risk of the endocervical margin being affected is 6.5 times greater (p=0.00002), while the risk of the ectocervical margin being affected is 6 times greater (p=0.00004). Gardeil et al.^14^ studied 225 women undergoing LEEP and found compromised margins in 105, with the endocervical margin affected in 72% and an incidence of recurrence of 16.5% in compromised margins and 1.9% in free ones.

The endocervical margin is generally more compromised in cases where the lesion enters the cervical canal or it is not possible to fully visualize the squamocolumnar Junction or if the lesion is extensive^3^ ^,^ ^11^ ^,^ ^14^ ^,^ ^20^ ^,^ ^22^.

Arbyn et al.^11^, in a review of 44,446 patients treated for CIN, stated that the margin status has a sensitivity of 55.8% and specificity of 84.4% for predicting recurrence.

A metanalysis by Ghaem-Maghami et al.^25^ with 27,785 cervix surgeries found 18% recurrence in compromised margins and 3% recurrence in free margins.

However, even with positive margins, due to the low recurrence rate, these patients do not need to be reoperated. According to Chen et al.^20^, the inflammatory reaction after the procedure can eliminate residual disease.

For better monitoring, molecular biology tests for HPV DNA have been used in clinical practice. Our work, on its turn, used immunomarkers (p16 and Ki-67) on surgical specimens to assess possible risk factors for recurrence.

p16 is a tumor suppressor protein belonging to the INK (cyclin-dependent-kinase) family. It has been used in cases of atypical squamous cells of undetermined significance (Ascus) and low-grade intraepithelial lesions on cytology. It has high sensitivity and specificity for high-grade lesions^6^ ^,^ ^7^ ^,^ ^26^ ^,^ ^27^.

Ki-67 is a marker of DNA proliferation and replication. In normal epithelium it is expressed in the basal and parabasal layers in the lower third of the epithelium, overexpression being associated with inflammation or atypia. The positive association of both can differentiate benign reactions, such as atrophy and metaplasia, from premalignat lesions. It is used for prognosis and prediction of tumors, as well as assisting in the diagnosis of cancer and pre-cancer^6^ ^,^ ^7^ ^,^ ^26^ ^,^ ^27^.

Our work did not show statistical significance of biomarkers in predicting cervical lesion recurrence.

Fonseca et al.^24^ did not find an association between p16 and recurrence, but the authors studied LEEP specimens with low- and high-grade lesions and adenocarcinoma, not excluding immunosuppressed patients, which may have altered the real reason for recurrence.

Leite et al.^28^ studied 68 patients undergoing LEEP, including cases of low-grade, high-grade and HIV-positive lesions, and concluded that there was no association between the biomarkers p16 and Ki-67 with lesion recurrence.

Low-grade lesions have a different evolution from high-grade ones, as most of the former regress spontaneously^29^. Therefore, analyzes of the results of low-grade lesions must be carried out separately from high-grade lesions’ ones, as well as the study of HIV+ patients, which present a higher risk of recurrence after treatment^30^.

Our study had some limitations, such as a retrospective design, which may have confounding variables, and loss of more than 50% of samples when choosing blocks that had insufficient material to perform the tissue microarray.

However, it evaluates factors for recurrence of cervical intraepithelial lesions in a population of women with high-grade intraepithelial lesions, who underwent surgery under the supervision of two gynecologists with extensive experience in surgical techniques and prospective immunohistochemical evaluation.

## CONCLUSION

Compromise of the endocervical margins remains the main risk factor for recurrence after the treatment of high-grade cervical intraepithelial lesions. Age, size of the colposcopic lesion, type of surgery, and immunomarkers p16 and Ki-67 were not identified as risk factors.
